# Identification of three extra-chromosomal replicons in *Leptospira* pathogenic strain and development of new shuttle vectors

**DOI:** 10.1186/s12864-015-1321-y

**Published:** 2015-02-15

**Authors:** Weinan Zhu, Jin Wang, Yongzhang Zhu, Biao Tang, Yunyi Zhang, Ping He, Yan Zhang, Boyu Liu, Xiaokui Guo, Guoping Zhao, Jinhong Qin

**Affiliations:** Department of Microbiology and Immunology, Institutes of Medical Science, Shanghai Jiao Tong University School of Medicine, 280 South Chongqing Road, Shanghai, 200025 China; CAS Key Laboratory of Synthetic Biology, Institute of Plant Physiology and Ecology, Shanghai Institute for Biological Sciences, Chinese Academy of Sciences, Shanghai, China; State Key Laboratory of Genetic Engineering, Department of Microbiology, School of Life Sciences, Fudan University, 220 Handan Road, Shanghai, 200433 China

**Keywords:** *Leptospira interrogans*, Plasmid, Prophage, Genetic transformation systems

## Abstract

**Background:**

The genome of pathogenic *Leptospira interrogans* contains two chromosomes. Plasmids and prophages are known to play specific roles in gene transfer in bacteria and can potentially serve as efficient genetic tools in these organisms. Although plasmids and prophage remnants have recently been reported in *Leptospira* species, their characteristics and potential applications in leptospiral genetic transformation systems have not been fully evaluated.

**Results:**

Three extrachromosomal replicons designated lcp1 (65,732 bp), lcp2 (56,757 bp), and lcp3 (54,986 bp) in the *L. interrogans* serovar Linhai strain 56609 were identified through whole genome sequencing. All three replicons were stable outside of the bacterial chromosomes. Phage particles were observed in the culture supernatant of 56609 after mitomycin C induction, and lcp3, which contained phage-related genes, was considered to be an inducible prophage. *L. interrogans–Escherichia coli* shuttle vectors, constructed with the predicted replication elements of single *rep* or *rep* combined with *parAB* loci from the three plasmids were shown to successfully transform into both saprophytic and pathogenic *Leptospira* species, suggesting an essential function for *rep* genes in supporting auto-replication of the plasmids. Additionally, a wide distribution of homologs of the three *rep* genes was identified in *L. interrogans* isolates, and correlation tests showed that the transformability of the shuttle vectors in *L. interrogans* isolates depended, to certain extent, on genetic compatibility between the *rep* sequences of both plasmid and host.

**Conclusions:**

Three extrachromosomal replicons co-exist in *L. interrogans*, one of which we consider to be an inducible prophage. The vectors constructed with the *rep* genes of the three replicons successfully transformed into saprophytic and pathogenic *Leptospira* species alike, but this was partly dependent on genetic compatibility between the *rep* sequences of both plasmid and host.

**Electronic supplementary material:**

The online version of this article (doi:10.1186/s12864-015-1321-y) contains supplementary material, which is available to authorized users.

## Background

Leptospirosis is one of the most widespread zoonoses caused by infection with a group of pathogenic spirochetes, primarily various strains of the species *Leptospira interrogans* [1,2]. Since the first complete genome of the bacterium was published a decade ago [3], remarkable progress has been made in understanding its genetic blueprint as well as the functions of a variety of its genes. However, major obstacles in the genetic analysis of *L. interrogans* remain; these are partly related to the slow growth rate of the bacterium and the lack of efficient genetic manipulation tools [4-6].

Plasmids and prophages are known to contribute to horizontal gene transfer, and their remnants are commonly found in bacterial genomes [7-11]. Because these elements can carry diverse genetic information allowing them to play specific physiological roles in the host bacterium, they potentially serve as efficient genetic tools [12-16]. Numerous linear and circular plasmids have been found in another pathogenic spirochete, *Borrelia burgdorferi*, and nine of them were identified as being prophage derived [17-21]. Among them, pGK12 was developed as an expression vector for *B. burgdorferi*, and this plasmid has significantly assisted investigation of the *in vivo* functions of particular genes [22-24].

In the genus *Leptospira*, saprophytic *Leptospira biflexa* serovar Patoc strain Patoc I was shown to harbor a plasmid (P74) and atemperate phage, LE1 [25-28]. The latter, which was developed as a *L. biflexa*–*Escherichia coli* shuttle vector, contains the LE1 replication region and an antibiotic resistance marker, and has been shown to replicate in saprophytic *L. biflexa*, but not in pathogenic *L. interrogans* [27]. Although plasmids and prophage remnants have recently been reported in other *Leptospira* species [29], a leptospiral genetic transform system has not yet been well established. Recently, random transposon mutagenesis and targeted mutagenesis in pathogenic leptospires have been achieved, and these have undoubtedly assisted genetic characterization of potential virulence factors in pathogenic *L. interrogans* [5,6,30-32]. A method for conjugative transfer between *E. coli* and *Leptospira* spp. has also been developed using RP4 derivative conjugative plasmids to deliver the transposon, *Himar1* [4]. However, genetic complementation of knockout mutants remains a challenge and still has to be conducted using transposon-mediated insertion or homologous recombination. Because the transposon integration site is uncertain, substantial time and energy are required to verify successful complementation. Therefore, development of an efficient genetic manipulation system in pathogenic *Leptospira* remains a highly warranted research goal.

In this study, we sequenced the complete genome of the highly virulent *L. interrogans* serogroup Grippotyphosa serovar Linhai strain 56609, followed by an intensive and detailed study of the three extrachromosomal circular replicons that were identified, with the goal of obtaining better understanding of this bacterium.

## Results

### Complete sequencing and *de novo* assembly of the *L. interrogans* serovar Linhai strain 56609 genome revealed three extrachromosomal circular DNA replicons

**Figure 1:**
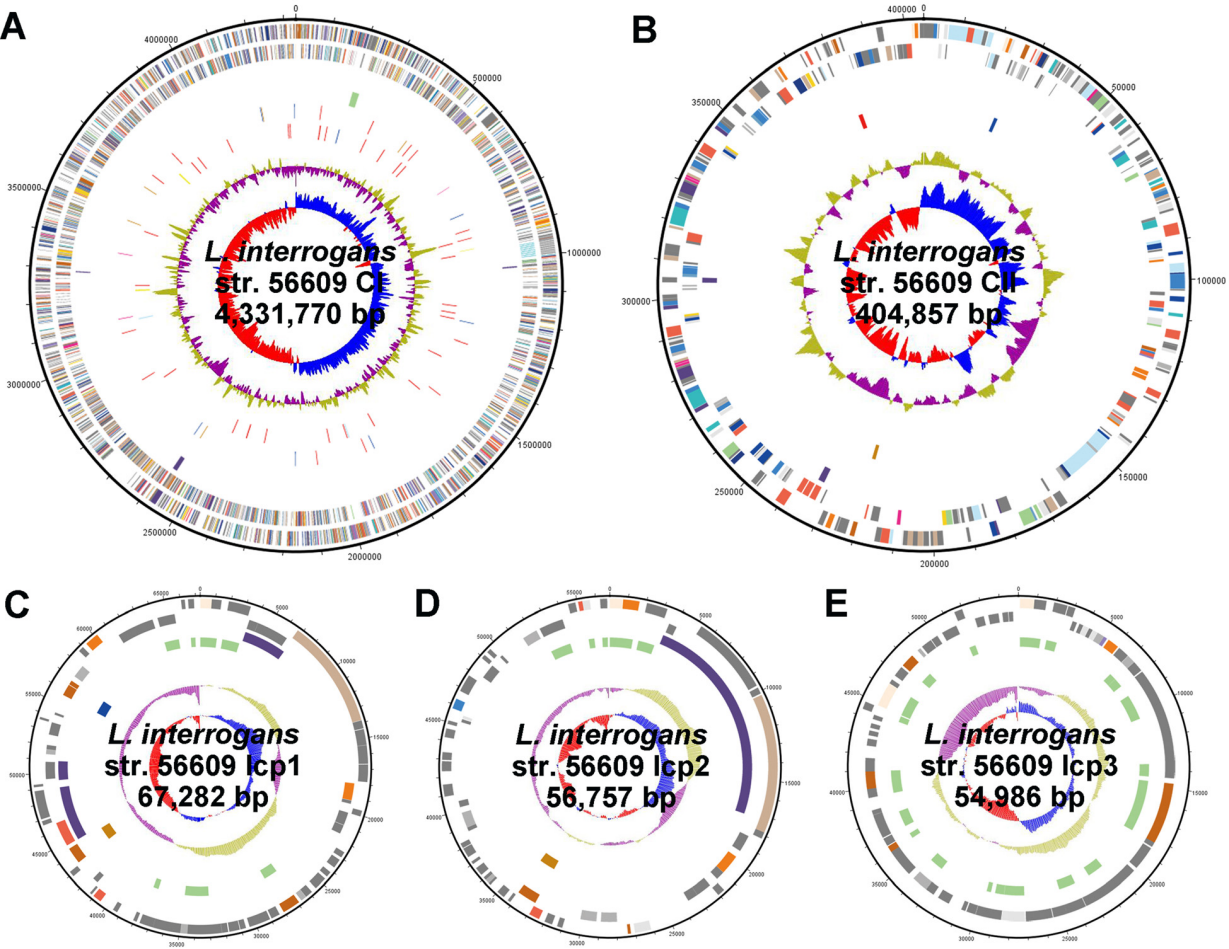
**Genome maps of** ***L. interrogans*** **strain 56609.** Circles are numbered from outer to inner and are designated as follows. **(A-B)** Large chromosome (CI) and small chromosome (CII), **(C-E)** Plasmid lcp1-3. Circles 1 and 2 denote forward and reverse strand genes (colors represent functional categories according to COGs). Circles 3, identical sequences between chromosomes and plasmids. Circles 4, phage related CDSs. Circles 5, IS element (both intact and remnant are included). Circles 6, tRNA genes and rRNA genes. The two inner circles for the chromosomes display GC content and GC skew calculated using a 10,000 bp (CI) / 5,000 bp (CII) window sliding 200 bp at a time. The two inner circles for the plasmids display GC content and GC skew calculated using a 5,000 bp window sliding 100 bp at a time. Identical sequences are listed in Additional file 1: Table S2.

Three circular extrachromosomal replicons, designated lcp1, lcp2, and lcp3 were found in *L. interrogans* serovar Lai strain 56609 through whole genome sequencing and *de novo* assembly (Figure 1). According to the level of sequence-read coverage in each assembly, it was estimated that the three replicons shared equal copy numbers with the chromosomes. The overall G + C contents of lcp1 and lcp2 were, at about 35%, similar to that of the chromosomes, whereas lcp3 had a relatively higher G + C content of 39%. Basic Local Alignment Search Tool (BLAST) analysis suggested that the vast majority of the genes encoded by the three replicons were hypothetical proteins, while a small portion encoded phage-related proteins (Additional file 1: Table S1). It is noteworthy that lcp3 encoded more phage-related proteins (22, 73.3% of the total 30 coding sequences with assigned functions) than the other two extrachromosomal replicons, lcp1 (9, 29.0% of the total 31 coding sequences with assigned functions) and lcp2 (6, 20% of the total 30 coding sequences with assigned functions). Furthermore, the strand-bias distribution of the lcp3 genes, with 71 out of 77 genes clustered on the same strand was also significantly different from that of the other two replicons.

### Plasmid DNA extraction followed by physical mapping confirmed the auto-replication traits of lcp1, lcp2, and lcp3

To confirm that the three putative low-copy-number circular plasmid replicons (lcp1, lcp2 and lcp3) were definitely extrachromosomal replicons, plasmid DNA from *L. interrogans* serovar Linhai strain 56609 was extracted using the classical alkaline lysis method and was then used for Southern blot analysis employing both plasmid-derived and chromosomal DNA-specific probes. As indicated in Additional file 2, in addition to the untreated DNA extracts, a specific restriction endonuclease, which has a unique restriction site in each of the candidate plasmids, was used to digest the DNA extracts separately in order to observe their linear configurations. DNA bands corresponding to different configurations of the plasmids were apparent after hybridization with plasmid-specific probes but absent using the chromosomal *flaB* specific probe. This result suggested that the three replicons were all present in the bacteria as circular extrachromosomal plasmids.

**Figure 2:**
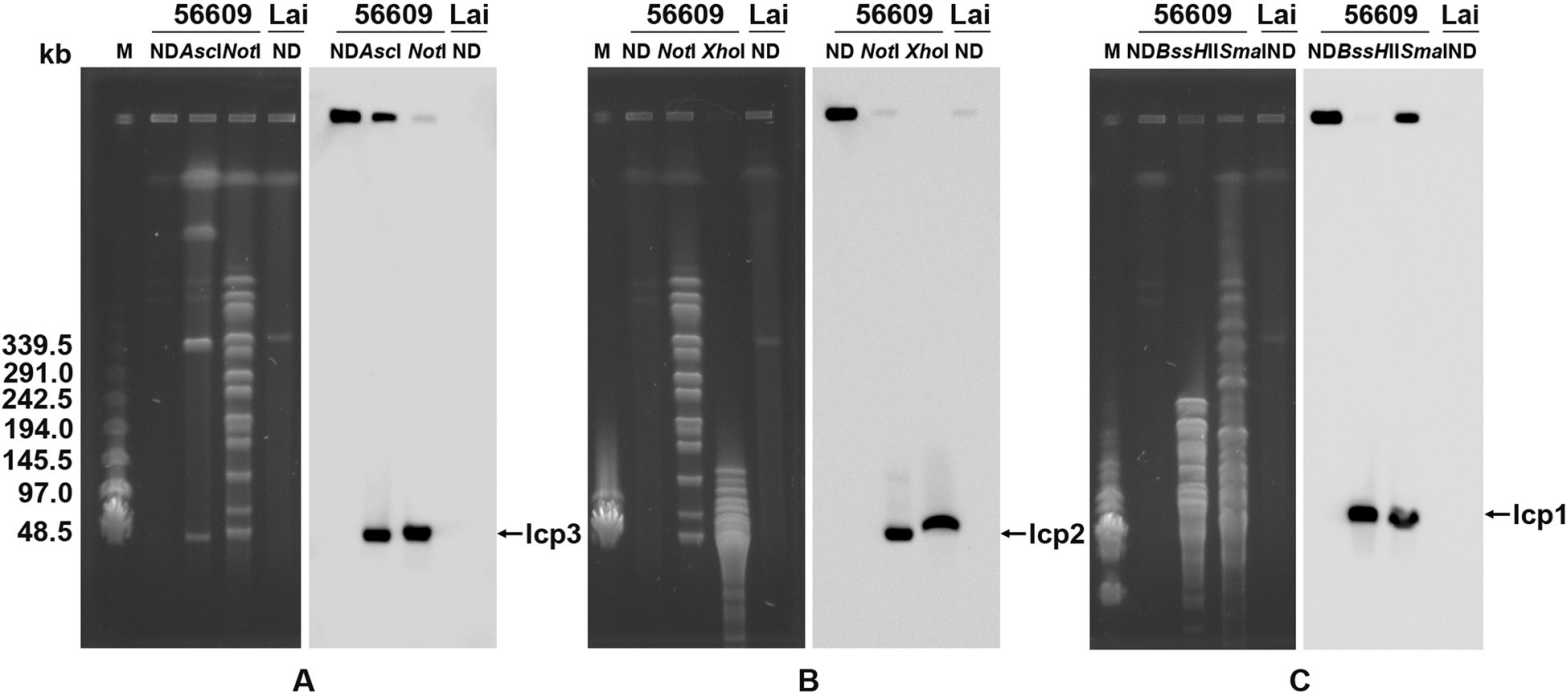
**Detection of the circular features of the three plasmids by PFGE. (A-C)** Plasmid detection by PFGE separation and Southern blot hybridization. **A**, hybridization with lcp3-specific probes after PFGE separation; **B**, hybridization with lcp2-specific probes after PFGE separation; **C**, hybridization with lcp1-specific probes after PFGE separation. The left side of each picture represents PFGE separation of genomic DNA while the right side represents Southern blot hybridization with plasmid-specific probes; M, bacteriophage λ DNA multimer marker (monomer =48.5 kb). ND, undigested DNA; *Asc*I, *Not*I, *BssH*II, *Sma*I, and *Xho*I represent *L. interrogans* strain 56609 digested with these enzymes. *L. interrogans* serovar Lai strain 56601 genomic DNA was also blotted onto a nylon membrane as a negative control. The primers for the synthetized probes are listed in Additional file 1: Table S3.

To determine whether the three plasmids were integrate into chromosomes, genomic DNA was digested with selected rare-cutting restriction enzymes followed by *in situ* pulsed-field gel electrophoresis (PFGE)-based Southern blot analysis. For lcp3, cutting with the restriction enzymes *Not*I and *Asc*I (each has a single restriction site in lcp3), was confirmed by Southern blotting using an lcp3-specific probe. The result showed that DNA bands of about 50 kb were obtained, a result consistent with the *in silico* predicted size of lcp3 (Figure 2A). Similarly, after digestion with a single restriction enzyme and Southern blot hybridization with lcp1- and lcp2-specific probes, the same restriction patterns were observed for lcp1 and lcp2; these were identical to the linearized forms of these plasmids (Figure 2B and C). When the extracted genomes containing the replicons were analyzed by PFGE without digestion, no linear forms of the replicons were detected by Southern blots, and the visible bands after ethidium bromide staining possibly represented remnants of the degraded genomes.

**Figure 3:**
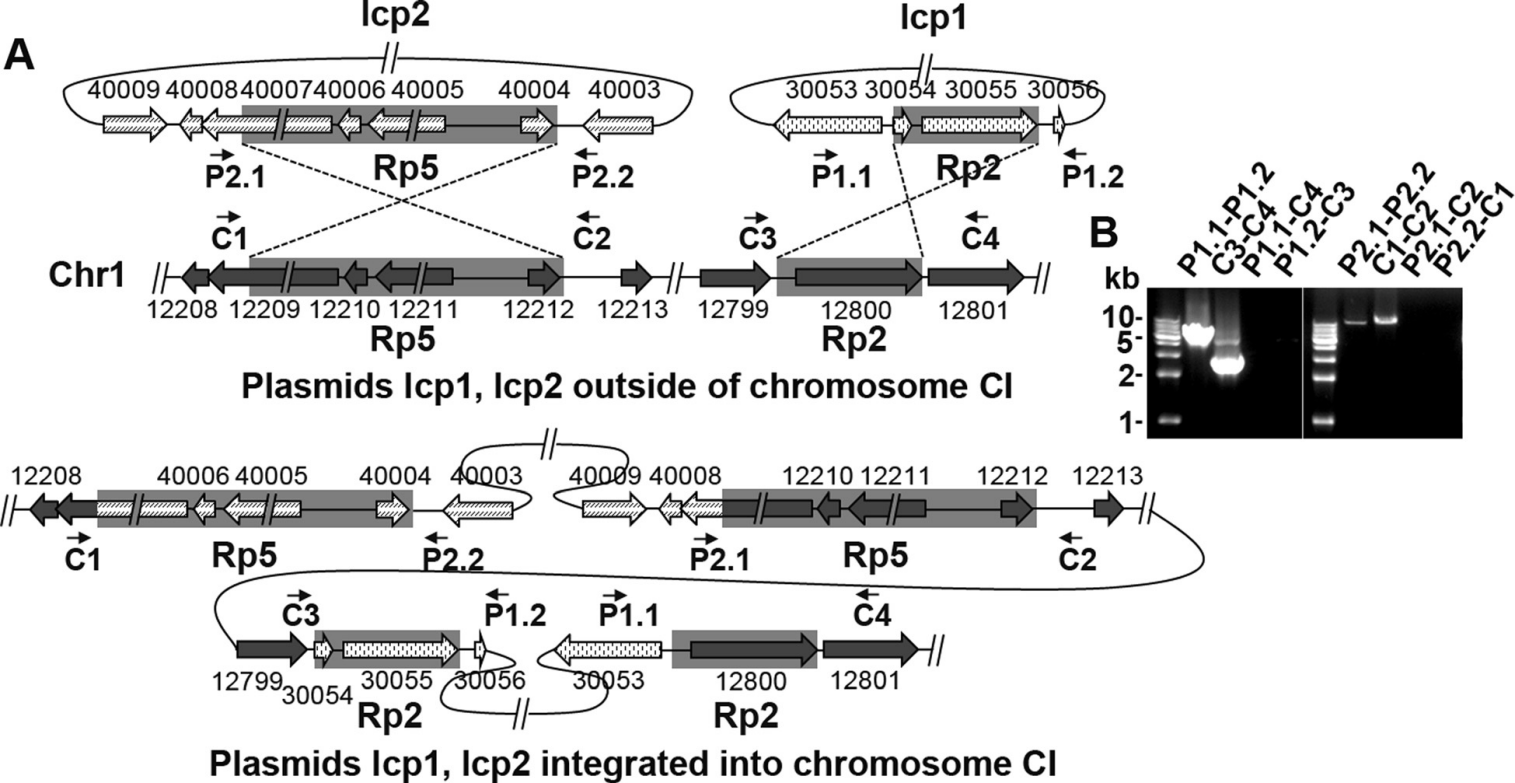
**Location relationship of lcp1 and lcp2 with chromosome CI. (A)** Schematic diagram of the two putative locations of lcp1 and lcp2 on chromosome CI based on sequence information. Identical sequences in chromosome CI and the plasmids are shown in gray shadow. The locations of the primers used for PCR amplification are indicated (“C” indicates chromosome, “P” indicates plasmid). The figure is drawn to scale. **(B)** PCR results confirming the plasmids are circular replicons outside of the chromosome CI. PCR amplification was performed with the primers indicated.

During sequence assembly, we found five sequences that were identical among the lcp1 and lcp2 plasmids and the chromosomes listed in Additional file 1: Table S2. To confirm the circular nature of lcp1 and lcp2, and to also exclude the possibility of plasmid integration, polymerase chain reaction (PCR) strategies employing primer pairs encompassing identical sequences between the plasmids and chromosomes were amplified, followed by DNA sequencing of the PCR products. Figure 3 shows the PCR results encompassing the large identical sequence repeats 5 (rp5) (13.7 kb) between lcp2 and chromosome CI as well as the identical repeat sequence rp2 (2.3 kb) between lcp1 and chromosome CI. PCR products were successfully obtained with the plasmid-located primer pairs (i.e. P2.1 and P2.2, P1.1 and P1.2), as were products from chromosomally located primer pairs (i.e. C1 and C2, C3 and C4), thereby confirming that lcp1 and lcp2 are extrachromosomal. The assumption that the two plasmids might be capable of chromosomal integration was rejected when no PCR products from plasmid-located primer and chromosomally located primer pairs were detected (e.g. P1.1 and C4, P1.2 and C3, P2.1 and C2, P2.2 and C1), thus excluding the possibility of lcp1 and lcp2 integrating into the chromosomes (Figure 3).

Taken together, these results show that the three plasmids are extrachromosomal, and also exclude the possibility that they might be able to integrate into chromosomes. In addition, PFGE analysis of strain 56609′, which originated from strain 56609 after prolonged *in vitro* laboratory passage, digested with same single restriction enzymes, showed identical bands as those from strain 56609, which further confirms the high stability of these circular plasmids (Additional file 3).

### Plasmid lcp3 is likely to be an inducible prophage in *L. interrogans strain* 56609

**Figure 4:**
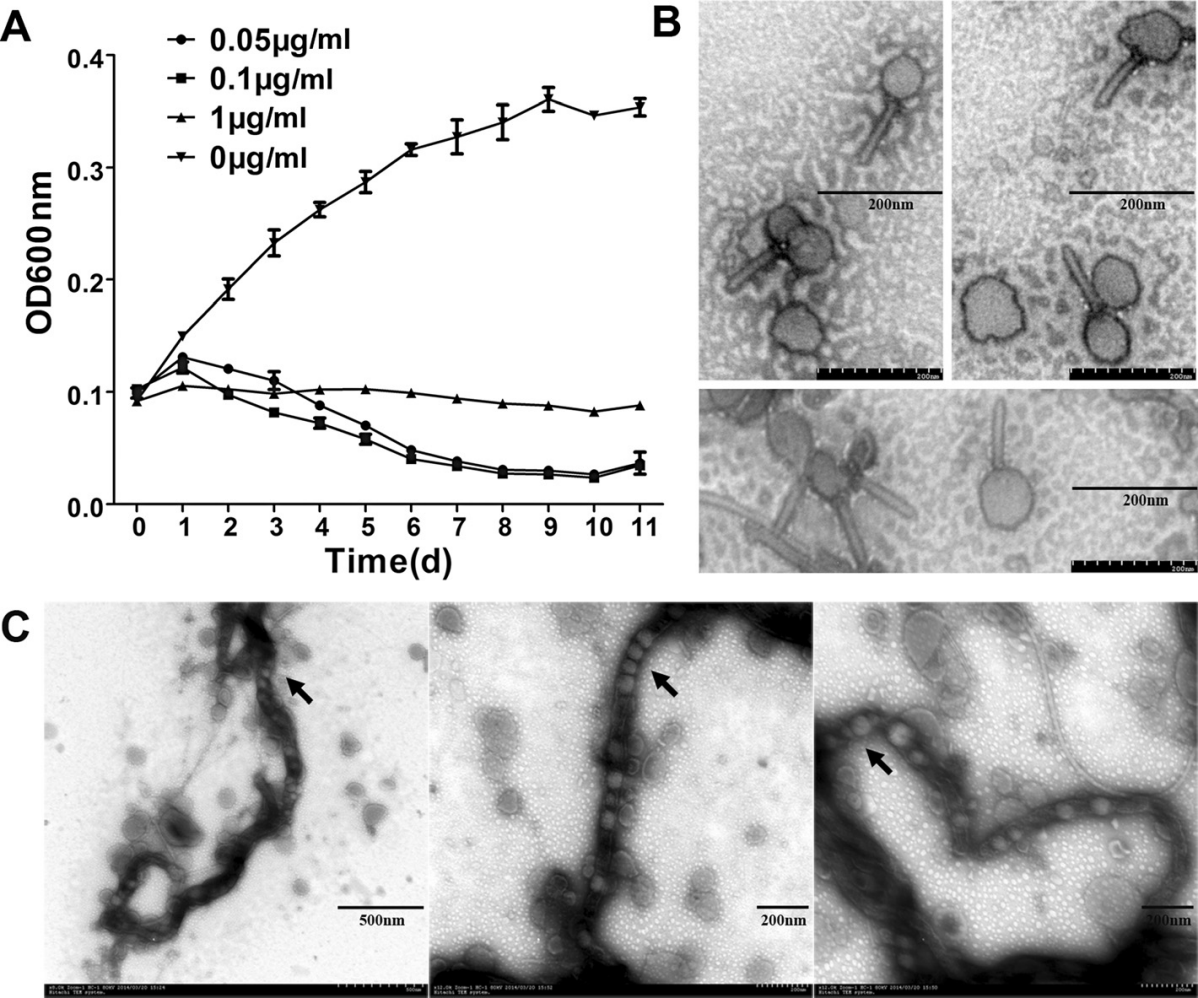
**Phage particle induction from** ***L. interrogans*** **strain 56609. (A)** *Leptospira* growth curve induced by different concentrations mitomycin C. Experiments were performed in triplicate. **(B)** Transmission electron microscopy photograph of phage particles. bar, 200 nm. **(C)** Transmission electron microscopy photograph of phage particles inside a *Leptospira* cell. bar (left), 500 nm, bar (middle and right), 200 nm.

According to the annotation results, plasmid lcp3 encodes 30 phage-related proteins, including those for bacteriophage replication, regulation, packaging, head and tail morphogenesis, and lytic cycle-related proteins; however, it lacks integrase and is, therefore, thought to be a prophage. Mitomycin C was used to induce prophage production. Two days after addition of mitomycin C to *L. interrogans* strain 56609 cultures, cell lysis was apparent by a decrease in the optical density of the culture (Figure 4A); lysis was nearly complete in 8 days, and was confirmed by dark-field microscopy. Transmission electron microscopy examination revealed bacteriophage-like particles (Figure 4B), each with a 45–50 nm diameter isometric head and an approximately 90-nm-long noncontractile tail, both of which are morphologically similar to those of the *Siphoviridae* family. Additionally, phage particles of the same size were observed inside *L. interrogans* cells 12 h after addition of mitomycin C (Figure 4C).

Expression of all 60 phage-related genes in lcp1, lcp2, and lcp3 was examined by reverse-transcription (RT)-PCR after mitomycin C induction. With the exception of the open reading frames (ORFs) *LIL50001* to *LIL50003*, which are involved in the partition and replication of plasmid lcp3 and are expressed at all stages, all of the other phage-related genes in lcp3 were specifically activated at different times by mitomycin C induction. Meanwhile, most ORFs in lcp1 and lcp2 showed no obvious changes in expression after mitomycin C induction (Additional file 4A). Seven representative genes in lcp3 were chosen for further examination by quantitative (q)PCR, and similar results were obtained (Additional file 4B). These results, to some extent, suggest that plasmid lcp3 is likely to be an inducible prophage in *L. interrogans* strain 56609.

### Characterization of replication origins in the three plasmids and development of a transformation system for pathogenic *Leptospira* species

Plasmid partition systems usually consist of the two partition proteins called ParA and ParB; these constitute the partition complex and are encoded by two coupled genes known as *parAB* [33,34]. Bioinformatic analysis revealed *parAB* genes in the three plasmids (i.e. *LIL30001* and *LIL30002* in lcp1, *LIL40001* and *LIL40002* in lcp2, and *LIL50001* and *LIL50002* in lcp3). Furthermore, *rep* genes encoding replication proteins in the three plasmids (i.e., *LIL30003* in lcp1, *LIL40003* in lcp2, and *LIL50003* in lcp3) were found to be located immediately downstream of *parAB* in their corresponding plasmids. All of these Rep proteins contain putative helix-turn-helix motifs, similar to Orf5 encoded by the LE1 phage genome in *L. biflexa* [27], and LA1839 encoded by the genomic island LaiGI in the *L. interrogans* serovar Lai genome [35]. LIL50003 in lcp3 exhibited low sequence similarity with Orf5 with only 27% identity and 73% coverage, whereas LIL40003 in lcp2 was highly similar to LA1839 with 96% identity and 96% coverage.

**Figure 5:**
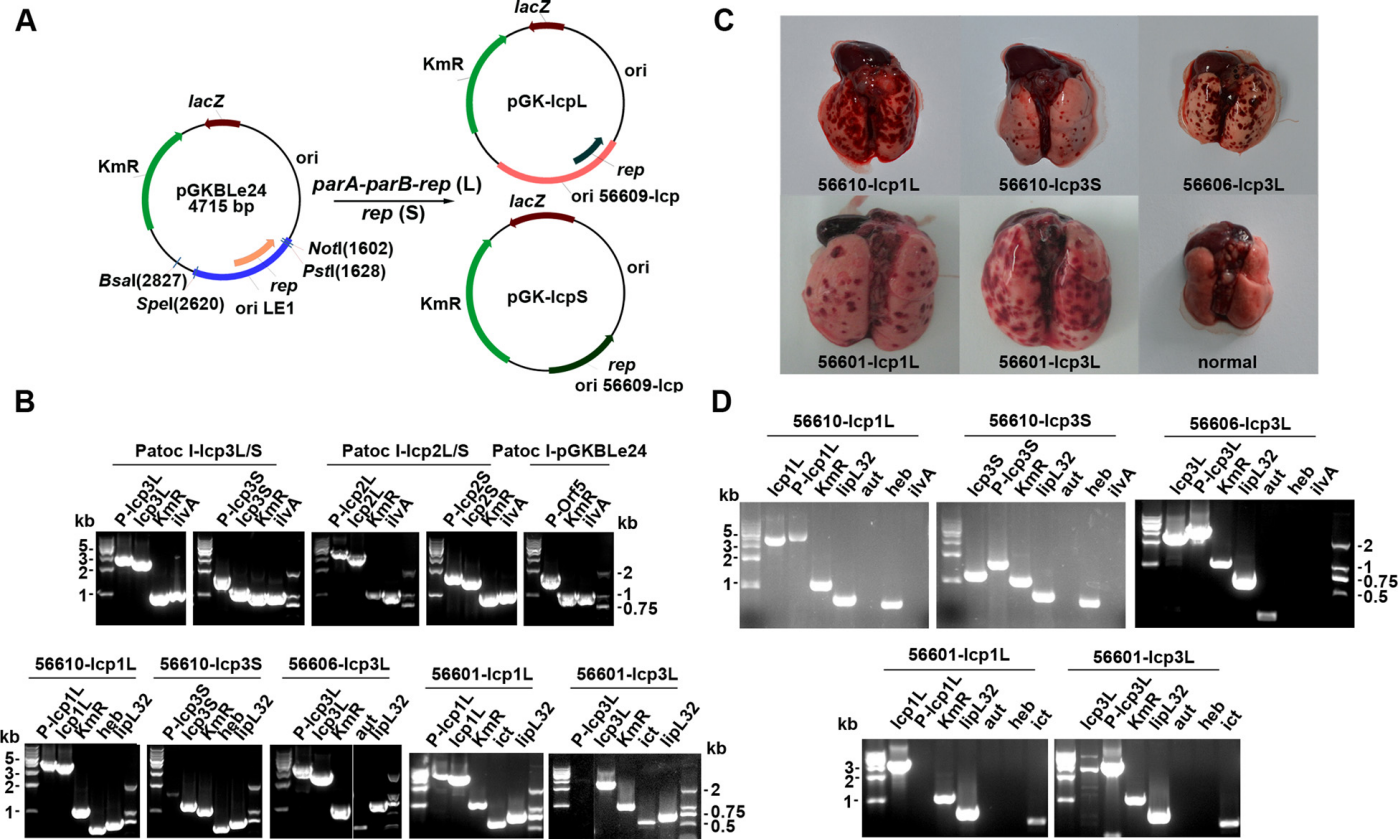
**Identification of the functional replication region within the three plasmids. (A)** Schematic diagram of the *L. interrogans–E. coli* shuttle vectors derived from pGKBLe24. KmR, kanamycin resistance cassette. Unique restriction sites used for shuttle vector construction are indicated. pGK-lcpL and pGK-lcpS are shuttle vectors containing the predicted *parA-parB-rep* and *rep* gene alone, respectively. **(B)** PCR-verified transformants. PCR amplification was performed using genomic DNA isolated from transformants that were picked from plates and enriched in liquid EMJH. **(C)** Lung lesion from a hamster infected with pathogenic *L. interrogans* transformants harboring the shuttle vectors. **(D)** PCR verification of pathogenic *L. interrogans* transformants recovered from liver. Primers are listed in Additional file 1: Table S3. Patoc I-lcp3L/S represents the confirmation of vectors lcp3L/S transformed into Patoc I strains. The same as for Patoc I-lcp2L/S, Patoc I- pGKBLe24, 56610-lcp1L, 56610-lcp3S, 56606-lcp3L, 56601-lcp1L, 56601-lcp3L. P-lcp3L is the PCR product from plasmid primers amplifying the fragment including the inserts. The same as for P-lcp3S, P-lcp2L, P-lcp2S, P-orf5, P-lcp1L. ilvA, primers for the specific gene *LEPBI_I1590* in *L. biflexa* serovar Patoc strain Patoc 1; heb, primers for the O-antigen-specific gene *heb* from the *L. interrogans* serogroup Hebdomadis strain 56610; aut, primers for the O-antigen-specific gene *aut* from the *L. interrogans* serogroup Autumnalis strain 56606; ict, primers for the O-antigen-specific gene *ict* from the *L. interrogans* serogroup Icterohaemorrhagiae strain 56601; lipL32, primers for the *L. interrogans* lipoprotein gene *lipL32* [56] of strain 56610, 56606 and 56601.

To characterize the essential replication regions in the three plasmids, DNA fragments containing single *rep* or *rep* combined with *parAB* loci were PCR amplified and then cloned into the *L. biflexa–E. coli* shuttle vector (pGKBLe24) to replace the original LE1 *rep* gene. Six *L. interrogans–E. coli* shuttle vectors were thus generated and designated lcp1L and lcp1S, lcp2L and lcp2S, and lcp3L and lcp3S, containing either *rep* and *parAB* in “L” or *rep* alone in “S”, respectively. The plasmids prepared from *E. coli* were transformed into different *Leptospira* strains by electroporation. Transformed strains included the pathogenic *L. interrogans* strains 56601, 56606 and 56610, and the saprophytic *L. biflexa* strain Patoc I. The LE1 *rep*-based shuttle vector pGKBLe24 was used as a positive control and its *rep* deleted pGKBLe24^-rep^ derivative was used as a negative control (Figure 5A). As expected, the positive control (pGKBLe24) was able to transform into saprophytic *L. biflexa* but was unable to transform into pathogenic *Leptospira* species [27]. Shuttle vectors lcp3L/S from lcp3 were able to transform into all four of the *Leptospira* strains we tested. However, the shuttle vectors lcp1L/S from lcp1, could transform into the 56601and 56610 strain, while the shuttle vectors lcp2L/S from lcp2 could only transform into the saprophytic *L. biflexa* Patoc I strain. All transformants were confirmed by PCR using primers specific for the shuttle vectors (Figure 5B). The plasmid transformation efficiencies for the saprophytic *L. biflexa* and pathogenic *L. interrogans* strains were similar, reaching 5 × 10^−6^ electrotransformants per recipient cell. The stability of the shuttle vectors in *L. interrogans* cells was confirmed by a hamster infection model and PCR amplification of the shuttle vector genes in pathogenic *L. interrogans* recovered from the livers of the infected hamsters (Figure 5C and D). Our data not only confirmed autonomous replication capability for all three plasmids, but also revealed the differential transformability of the shuttle vector constructs with different replication origins against various kinds of recipient hosts, as summarized in Table 1.

**Table 1:** **Transformation efficiency of shuttle vectors constructed in this study in four Leptospira strains**

**Leptospira strains**	**lcp1L/S**	**lcp2L/S**	**lcp3L/S**	**pGKBLe24** ^**-rep**^			**ddH**_**2**_ **O**	**pGKBLe24**
				**lcp1**	**lcp2**	**lcp3**		
*L. interrogans* serovar Autumnalis str. 56606	-	-	+	-	-	-	-	-
*L. interrogans* serovar Hebdomadis str. 56610	+	-	+	-	-	-	-	-
*L. interrogans* serovar Lai str. 56601	+	-	+	-	-	-	-	-
*L. biflexa* serovar Patoc str. Patoc I	-	+	+	-	-	-	-	+
*L. interrogans* serovar Linhai str. 56609	-	-	-	-	-	-	-	-

To detect if differences in host-specificity resulted from genetic incompatibility in the hosts, we tested 15 *Leptospira* strains endemic in China for the presence of *rep* genes from the three plasmids using Southern blot hybridization (Additional file 5). Comparisons of the transformability data against the *rep* sequence distribution for the three plasmids from strains 56601, 56606 and 56610 showed that the shuttle vectors only transformed hosts lacking the *rep* sequence of the same origin. Therefore, genetic incompatibility is likely to account for the differences observed in the shuttle vectors abilities to transform into different *Leptospir*a species recipients.

Finally, *Rep* protein from the three plasmids studied herein appears to be widely distributed among *Leptospira* strains, as shown by NCBI nonredundant protein database searches on *Leptospira* species genome using BLASTp, where 49 hits for lcp1, 22 hits for lcp2, and seven hits for lcp3 (at least 75% query coverage and 75% identity) were obtained. This finding again suggests that complete sequencing and *de novo* assembly are critical for novel discoveries from genomic data mining.

## Discussion

Extrachromosomal replicons were identified in pathogenic *L. interrogans* in this study. Because of the high proportion of homologous sequences among lcp1, lcp2 and chromosomes, plasmids tend to be easily assembled into chromosomes. Additionally, the low copy number of plasmids makes it hard to differentiate them from the large number of sequence reads generated from chromosomes. Furthermore, low sequence similarity among proteins encoded by extrachromosomal replicons also makes it difficult to identify plasmid-specific proteins based on sequence comparisons against other well-characterized plasmids. Also, the large sizes of plasmids also challenges methods designed to verify auto-replicons in bacteria. In this study, we confirmed the location of all three replicons in *L. interrogans* through the combined use of genomic sequence-directed PCR, plasmid-specific probe-based Southern hybridization and extrachromosomal replicon-oriented PFGE. All the results indicated the stable presence of the three plasmids as auto-replicons in the bacterium. Furthermore, we observed phage particles in *L. interrogans* strain 56609 cultures by induction with mitomycin C, a well-known inducer of phages. Genomic annotation identified *parA*, *parB*, and *rep* homologous sequences in lcp1, lcp2, and lcp3, all of which are notable for their indispensable roles in plasmid replication and stability [33,34,36,37]. The function of the three replicons was verified also by constructing functional shuttle vectors capable of autonomous replication in *L. biflexa* and/or *L. interrogans*. The results confirmed that these replicons are extra-chromosomally present and able to be auto-replicated.

For the purpose of genetic manipulation, electroporation employing suicide delivery vectors is the first choice for most researchers. When transposons are constructed in a suicide delivery vector, the transformation efficiency is about 5.1 × 10^−9^ electrotransformants per recipient cell for *L. interrogans* and 2 × 10^−7^ for *L. biflexa* [4]. Conjugative plasmids delivering the Himar1 transposon between *E. coli* and *Leptospira* spp*.* reached efficiencies of 8.5 × 10^−8^ transconjugants per recipient cell in *L. interrogans* and 1 × 10^−6^ in *L. biflexa*, respectively, values around 10 times higher than that of transformation. Although these established genetics systems facilitate the manipulation of functional genes in *Leptospira,* particularly in saprophytic *L. biflexa*, genetic manipulation in pathogenic *L. interrogans* remains especially difficult because of the low efficiencies of electroporation and conjugation combined with the slow growth rate of *L. interrogans*. In this study, the electroporation transformation efficiency of the replication plasmids reached 5 × 10^−6^ electrotransformants per recipient cell in both *L. biflexa* and *L. interrogans.* This indicates that the transformation efficiency of these newly constructed replicative plasmids in *L. interrogans* is 100 to 1000 times higher than before.

Combining the transformability data from the shuttle vectors (lcp1L/S, lcp2L/S and lcp3L/S), with the wide distribution of the *rep* gene in different hosts, suggests that genetic incompatibility between plasmids and hosts should be considered in *Leptospira* strains because shuttle vectors apparently cannot transform into a host that harbors the same *rep* gene. However, a plasmid’s inability to be transformed cannot be fully attributed to genetic incompatibility between itself and its host. Although Orf5 is absent in *L. interrogans*, it is reported that a shuttle vector containing Orf5 is unable to transform into pathogenic *Leptospira* strains [27,28,35]. We have also confirmed that it was unable to transform into *L. interrogans* strains 56601, 56606 and 56610. It has been reported that the shuttle vector derived from the Rep protein LA1839 in LaiGI can transform into *L. interrogans* Hond Utrecht IV and *L. biflexa* Patoc I strains but is unable to transform the *L. interrogans* Copenhageni strain [35]. Meanwhile, the *rep* gene *LA_1839* was found to be present in the *L. interrogans* Hond Utrecht IV strain but not in the *L. interrogans* Copenhageni strain. Hence, further systematic analyses are needed to elucidate the mechanism(s) underlying genetic transformability among *Leptospira* species, especially for pathogenic *L. interrogans* strains.

## Conclusions

Research on pathogenic *Leptospira* has lagged far behind that of other pathogenic bacteria, primarily because of the lack of effective genetic manipulation tools suitable for *L. interrogans*. In fact, the first major breakthrough in understanding the genetics of *L. interrogans* came with the availability of its whole genome sequence [3,38,39] and accumulation of abundant genomic sequencing data from it. With comprehensive information about the genetic blue print of *L. interrogans* coupled with epigenetic readouts from transcriptomics and proteomics in this species, *in vitro* biochemical and molecular biological analyses have already unveiled some of the molecular mechanisms underlying the physiology and pathology of this bacterium. However, without access to an efficient genetic manipulation system for *in vivo* analysis, it is impossible to obtain comprehensive knowledge about the biology of *L. interrogans* through *in vitro* biochemical analysis and sequence data based on *in silico* analysis alone. Therefore, the shuttle vectors developed and validated in this study represent a new milestone in research on pathogenic *Leptospira*. Moreover, these resources have strong potential to improve our understanding of the genomic composition of *L. interrogans* and genetic incompatibility in this bacterium.

## Methods

### Bacteria

*L. interrogans* serogroup Grippotyphosa serovar Linhai strain 56609 was initially isolated from a human patient with leptospirosis in Linhai, Zhejiang Province in 1954, and was maintained by the Chinese Center for Disease Control and Prevention (China CDC). Its virulence was preserved by serial passages in guinea pigs. The corresponding avirulent strain 56609’ is derived from strain 56609 and has lost its virulence after more than 5 years of *in vitro* passages in our laboratory. Saprophytic *L. biflexa* serovar Patoc strain Patoc I, pathogenic *Leptospira interrogans* serovar Lai strain 56601, *L. interrogans* serovar Autumnalis strain 56606 and *L. interrogans* serovar Hebdomadis strain 56610 were also from China CDC. All the strains were grown in liquid Ellinghausen–McCullough–Johnson–Harris (EMJH) medium under aerobic conditions at 28°C, cultured to mid-exponential phase, and then collected for further analysis.

### Sequencing and assembly

Five micrograms of 56609 genomic DNA was used for preparing sequencing libraries according to the manufacturer’s protocol (454 Life Sciences, Roche). Sequencing was performed separately on two different 454 GS system platforms, one on the 454 GS 20 system and the other on the 454 GS FLX system [40-42]. In total, 554,443 reads were generated and 530,080 reads of high quality (96%) were selected for genome assembly, providing 29.5 fold coverage. Phred, Phrap, and Consed programs were used for quality assessment and sequence assembly [43-45]. Altogether, we obtained 295 contigs (235 contigs > 500 bp), and the N50 size of the contigs was 20,058 bps. Contig order was determined by ContigScape plugin [46] and by referring to the reference genome sequence of the *L. interrogans* serovar Lai strain 56601 and the serovar Copenhageni strain L1-130. Physical gaps were closed by primer walking, PCR, and multiplex PCR methods. Finally, the error rate for strain 56609 was 0.4 errors per 10,000 bps. Genome validation was achieved by comparison of the *in silico* digestion results with *in situ* PFGE using rare-cutting restriction enzymes. The complete genomic sequences of *L. interrogans* strain 56609 have been deposited in GenBank under the following accession numbers: CP006723, CP006724, CP006725, CP006726, and CP006727.

### Genome annotation

ORFs were identified by Glimmer, GeneMark, and Z-curve programs with a further manual check applied [47-49]. Functional annotation was performed by comparison with *L. interrogans* serovar Lai and Copenhageni reference strains through the BLASTp program using the blast search criterion E-value of 10^−3^, whereby at least 75% of the smaller sequence was aligned. Clusters of orthologous groups (COG )functional classification for each gene was assigned using RPS-BLAST against the Conserved Domain Database (CDD), and domain analysis was performed by searching the Pfam database with an E-value of 10^−3^, which gave further verification and supplementation of the annotation [50,51]. If there were no BLAST hits, another BLASTp search was performed against the NCBI nonredundant protein database to determine the sequence similarity by removing aligned regions that were less than 75% of the smaller sequence. Transfer RNA genes were identified with tRNAscan-SE [52]. Insertion sequence (IS) elements were found by searching with the IS-finder online tool (https://www-is.biotoul.fr/) and by BLAST searches according to the reference sequences.

### DNA manipulation

The classical alkaline lysis method was performed (with slight modification) to isolate large circular single-copy plasmids from *L. interrogans* strain 56609. *Leptospira* cells were resuspended in solution 1 (50 mM glucose, 10 mM EDTA pH 8.0, 25 mM Tris–HCl pH 8.0, 2.5 mg/ml lysozyme) at 37°C for 1 h. Solution 2 (0.2 N NaOH, 1% SDS) was added and the solution incubated at 55°C for 30 min. Solution 3 (5 M sodium acetate, 60 ml; acetic acid, 11.5 ml; sterilized water, 28.5 ml per 100 ml) was added with thorough mixing, and then the solution was cooled on ice for 5 min. After centrifugation, the supernatant was collected and extracted with acidic phenol–chloroform (NaAc-saturated phenol, pH 4.5: chloroform: isoamyl alcohol at 25: 24: 1) followed by pH neutral phenol–chloroform (Tris–HCl saturated phenol, pH 7.9: chloroform: isoamyl alcohol at 25: 24: 1). DNA was precipitated with isopropanol and washed twice with 70% ethanol. The purified DNA was dissolved in sterile deionized water and stored at −20°C.

For PFGE, the cells were embedded in agarose plugs and then lysed by protease treatment as previously described [53]. For digestion of DNA plugs, PFGE was performed in a contour-clamped homogeneous electric field DRII apparatus (Bio-Rad Laboratories). A program with ramping from 5 to 65 s for 20 h at 6 v/cm was employed to separate the DNA fragments. For Southern blot analysis, the digoxigenin-labeled probes were generated through PCR amplification using the oligonucleotide primers listed in Additional file 1: Table S3 (PCR DIG Probe Synthesis Kit, Roche). Hybridization and detection were performed with a DIG DNA detection system (Roche) according to the manufacturer’s protocol.

### Template prophage induction and microscopy of phage particles

A bacterial culture at early logarithmic growth phase was divided into four aliquots and a mitomycin C gradient at a final concentration of either 1 μg/ml, 100 ng/ml, 50 ng/ml, or 0 ng/ml, was added to each aliquot. Phage particles were collected after 4 days with 100 ng/ml mitomycin C added by centrifugation at 12,000 × g for 30 min twice to remove the residual bacteria followed by a final ultracentrifugation at 40,000 × g for 2 h. The phage sample was stained with 2% phosphotungstic acid and examined on a Hitachi 700 transmission electron microscope.

### RNA isolation, reverse transcriptase polymerase chain reaction (RT-PCR), and quantitative PCR (qPCR)

Induced bacterial cells were harvested at various times after addition of 100 ng/ml of mitomycin C. RNA was extracted using TRIzol reagent (Invitrogen) according to the manufacturer’s instructions and converted to cDNA using the Transcriptor First Strand cDNA Synthesis Kit (Roche). PCR primer pairs were designed for target ORFs in the three plasmids. Seven genes from lcp3 were selected for qPCR. For each amplification reaction, the C_*T*_ (threshold cycle) of the *flaB* gene from the same sample was used as an internal control to normalize the tested gene amplicon for C_*T*_ calculation, and the relative fold changes were calculated as described previously [54,55].

### Plasmid construction and electroporation-mediated transformation of *Leptospira* species

The nucleotide sequences of the three plasmid replication regions (single *rep* and *rep* combined with the *parAB* locus) were amplified with the primer pairs shown in Additional file 1: Table S3 and inserted into the *L. biflexa–E. coli* shuttle vector pGKBLe24 (a gift from Dr. Picardeau, Institute Pasteur, France) to replace the LE1 replication region and generate new plasmids, herein referred to as pGK-lcpL and pGK-lcpS. The pGKBLe24^-rep^ (LE1 replication region deletion with different restriction enzyme sites) of each three plasmids was self-ligated as a negative control. The new shuttle vectors and control plasmids were enriched from *E. coli* using a Plasmid Maxi Kit (QIAGEN). *Leptospira* cells were electrotransformed as described previously [27]. Kanamycin-resistant clones were picked from plates and transferred to 2 ml EMJH liquid media containing kanamycin and cultured until they had grown sufficiently. Further verification was achieved by genomic DNA isolation followed by PCR amplification with the primers listed in Additional file 1: Table S3 [56].

## Additional files

Additional file 1: Table S1. General feature of *L. interrogans* serovar Linhai str. 56609 genome. Table S2. Identical sequences between plasmids lcp1 and lcp2 and chromosomes*. Table S3 Oligonucleotide primers used in this study. Table S4. Distribution of three plasmids in 15 Chinese epidemic Leptospira strains. Additional file 2:
**Characterization of genome DNA from the three plasmids by agarose gel electrophoresis and Southern blotting.**
Additional file 3:
**Stability of the three plasmids within the** ***L. interrogans*** **strain 56609.**
Additional file 4:
**RT-PCR and qPCR to detect gene expression after induction.**
Additional file 5:
**Identification of the three plasmids in 15 Chinese epidemic Leptospira strains and the saprophytic** ***L. biflexa*** **serovar Patoc strain Patoc I.**
