# Supplementary material for: Identification of three extra-chromosomal replicons in Leptospira pathogenic strain and development of new shuttle vectors
Source: BMC Genomics. 2015 Feb 15;16(1):90. doi: 10.1186/s12864-015-1321-y (PMC4338851; doi:10.1186/s12864-015-1321-y)
Supplement: Additional file 1: Table S1. — General feature of L. interrogans serovar Linhai str. 56609 genome. Table S2. Identical sequences between plasmids lcp1 and lcp2 and chromosomes*. Table S3 Oligonucleotide primers used in this study. Table S4. Distribution of three plasmids in 15 Chinese epidemic Leptospira strains. [file 12864_2015_1321_MOESM1_ESM.docx]

**Table S1. General feature of *L. interrogans* serovar Linhai str. 56609 genome**

| Features of str. 56609 | CI | CII | lcp1 | lcp2 | lcp3 |
| --- | --- | --- | --- | --- | --- |
| Genome Size (bp) | 4,331,770 | 404,857 | 67,282 | 56,757 | 54,986 |
| G+C content (%) | 34.97 | 35.14 | 35.91 | 34.67 | 39.43 |
| Protein coding (%) | 75.8 | 78.0 | 75.4 | 68.8 | 83.9 |
| Total CDSs | 3,600 | 337 | 71 | 56 | 77 |
| CDSs with assigned function | 1,946 | 197 | 31 | 30 | 30 |
| Phage related CDSs (%)* | 30 (1.5) | 0 | 9 (29.0) | 6 (20) | 22 (73.3) |
| CDSs without assigned function | 1,654 | 140 | 40 | 26 | 47 |
| Average CDS length (bp) | 912 | 937 | 714 | 698 | 599 |
| CDS on forward strand | 1,861 | 189 | 49 | 37 | 6 |
| CDS on reverse strand | 1,739 | 148 | 22 | 19 | 71 |
| Total IS element | 29 | 3 | 2 | 1 | 0 |
| IS1500 | 5 | 1 | 1 | 1 | 0 |
| IS1501 | 7 | 1 | 1 | 0 | 0 |
| IS1533 (W) | 3 | 0 | 0 | 0 | 0 |
| ISLin1 | 14 | 1 | 0 | 0 | 0 |
| Transfer RNA | 37 | 0 | 0 | 0 | 0 |
| Ribosomal RNA | 5 | 0 | 0 | 0 | 0 |

*****Ratio of phage related CDSs in all CDSs with assigned function

**Table S2. Identical sequences between plasmids lcp1 and lcp2 and chromosomes***

| Name | Length | Location (bp) | | | |
| --- | --- | --- | --- | --- | --- |
|  |  | lcp1 | lcp2 | Chr1 | Chr2 |
| rp1 | 3.3kb | 3,466-6,726 |  | 1,024,366-1,027,627 |  |
| rp2 | 2.3kb | 46,612-48,898 |  | 3,289,482-3,291,766 |  |
| rp3 | 1.9kb | 44,719-46,609 |  |  | 232,469-234,358 |
| rp4 | 1.4kb | 49,453-50,881 |  |  | 304,253-305,682 |
| rp5 | 13.7kb |  | 3,488-17,208 | 2,560,930-2,574,651 |  |

*****Not including transposases; rp represents repeats

**Table S3 Oligonucleotide primers used in this study**

| **Oligonucleotide primers of probes** | | | |
| --- | --- | --- | --- |
| Primer | Sequence of primer (5'-3') | Location (bp) | |
| lcp1-probe-F | ATGGCCGAAAAAAATAAACT | 737-756 (lcp1) | |
| lcp1-probe-R | TTATAGATGTTTGAGATCGCTT | 1,345-1,366 (lcp1) | |
| lcp2-probe-F | CTAAGAGAATTGGCTAAAT | 55,256-55,274 (lcp2) | |
| lcp2-probe-R | TTACCGAAAATAGACCATA | 55,936-55,954 (lcp2) | |
| lcp3-probe-F | ATTTATGTCCGCACCGTG | 1,781-1,798 (lcp3) | |
| lcp3-probe-R | CATGTAAATCTACCTCGA | 2,564-2,581 (lcp3) | |
| flaB-probe-F | CATTGCCGTACCACTCTGT | 2,366,195-2,366,213 (CI) | |
| flaB-probe-R | GACGATGAAAGCTCTGTCT | 2,366,897-2,366,915 (CI) | |
| lcp1-rep-probe-F | GTCGTACATTACGCTTTGGT | 1,845-1,864 (lcp1) | |
| lcp1-rep-probe-R | CTTTACTCCCCACTTGTGTGA | 2,596-2,616 (lcp1) | |
| lcp2-rep-probe-F | GGAAGGTATCTATCTACGGA | 2,027-2,046 (lcp2) | |
| lcp2-rep-probe-R | CTGTACAGTAAACCCCACT | 2,486-2,504 (lcp2) | |
| lcp3-rep-probe-F | GTAGGCGAGTTTATACCTCGT | 1,829-1,849 (lcp3) | |
| lcp3-rep-probe-R | CTCAGAGATTTAGAGACTGGA | 2,410-2,430 (lcp3) | |
| **Oligonucleotide primers used for determination three plasmids organization** | | | |
| Primer | Sequence of primer (5'-3') | Location (bp) | |
| P1.1 | GCATCACTTTATAGATCTGAGGATA | 46,158-46,182 (lcp1) | |
| P1.2 | TTCAAATAGATTCGAGACTAGGAC | 54,971-54,994 (lcp1) | |
| C3 | GAACTTCCCACAGACTGGTAT | 3,289,438-3,289,458 (CI) | |
| C4 | GCAGATTTTGTTTTCTCTTCAGAT | 3,292,140-3,292,163 (CI) | |
| P2.1 | CCACTTTTATTACCTTTGTCAAGA | 17,484-17,507 (lcp2) | |
| P2.2 | TTAACTTGAATTTGGTATAAGGGA | 3,223-3,246 (lcp2) | |
| C1 | CAAGTTGAGAACCATAACCAGTAA | 2,560,704-2,560,727 (CI) | |
| C2 | CAATTGAGTCTATGACACCAGAGT | 2,574,672-2,574,695 (CI) | |
| **Oligonucleotide primers for shuttle vectors construction and transformants confirmation** | | | |
| Primer | Sequence of primer (5'-3') | | Location (bp) |
| lcp1-S-F | CTAACCTTTACCATGCGTCA | | 1,777-1,796 (lcp1) |
| lcp1-S-R | ATTGTTAGCCTCAAAGAT | | 3,593-3,610 (lcp1) |
| lcp1-L-F | GAGAATTTACAATGCATAAGT | | 67,128-67,148 (lcp1) |
| lcp1-L-R | ATTGTTAGCCTCAAAGAT | | 3,593-3,610 (lcp1) |
| lcp2-S-F | TAATAATTTAGTTATGT | | 1,774-1,790 (lcp2) |
| lcp2-S-R | TATAGTTCTACTCAACCA | | 3,403-3,420 (lcp2) |
| lcp2-L-F | TACTTACTAGTTTCTTTA | | 56,734-56,751 (lcp2) |
| lcp2-L-R | TATAGTTCTACTCAACCA | | 3,403-3,420 (lcp2) |
| lcp3-S-F | CTCAGGTCAAATTTGACA | | 1,385-1,402 (lcp3) |
| lcp3-S-R | CATGTAAATCTACCTCGA | | 2,564-2,581 (lcp3) |
| lcp3-L-F | TCGGCAGCTTGGTATGAT | | 54,860-54,877 (lcp3) |
| lcp3-L-R | TCGCCGACTGCATAAATA | | 2,584-2,601 (lcp3) |
| pGKBLe24-F | CTACGATACGGGAGGGCT | | 1,408-1,425 (pGKBLe24) |
| pGKBLe24-R | TCTGGAGCCGGTGAGCGT | | 2,839-2,856 (pGKBLe24) |
| KmR-F | TCGGCTCCGTCGATACTATG | | 3,244-3,263 (pGKBLe24) |
| KmR-R | CATCAGAGTATGGACAGTTGC | | 4,284-4,304 (pGKBLe24) |
| lipL32-F | ACTCTTCAGCAGCGATAGCT | | 1,740,372-1,740,391 (CI) |
| lipL32-R | GTTGCACTCTTTGCAAGCAT | | 1,741,106-1,741,125 (CI) |
| ilvA-F | GTGAAGACCTCCAAGTGGTT | | 1,666,275-1,666,294 (Patoc I CI) |
| ilvA-R | TATTCTTGCATACGGATGAG | | 1,667,315-1,667,334 (Patoc I CI) |
| aut-F | TTTTGATGGGCATACTGA | |  |
| aut-R | TATGCCCTAAGTGAGTTGC | |  |
| heb-F | GATTTGATAAGGCGAAGA | |  |
| heb-R | AAGCTCCAATACATAAGGAC | |  |

**Table S4. Distribution of three plasmids in 15 Chinese epidemic Leptospira strains**

| Strain | Species | Serovar | lcp1  Identity (%) | lcp2  Identity (%) | lcp3  Identity (%) |
| --- | --- | --- | --- | --- | --- |
| 56601 | *L.interrogans* | Lai | - | 99 | - |
| 56602 | *L.borgpetersenii* | Javanica | - | - | - |
| 56603 | *L.interrogans* | Canicola | - | - | - |
| 56604 | *L.borgpetersenii* | Ballum | - | - | - |
| 56605 | *L.interrogans* | Pyrogenes | 84 | - | - |
| 56606 | *L.interrogans* | Autumnalis | 99 | 99 | - |
| 56607 | *L.interrogans* | Australis | 85 | - | 97 |
| 56608 | *L.interrogans* | Pomona | - | - | - |
| 56609 | *L.interrogans* | Linhai | 99 | 99 | 99 |
| 56610 | *L.interrogans* | Hebdomadis | - | 99 | - |
| 56612 | *L.interrogans* | Paidjan | 96 | 99 | - |
| 56613 | *L.borgpetersenii* | Tarassovi | - | - | - |
| 56615 | *L.weilii* | Qingshui | - | - | - |
| 56635 | *L.interrogans* | Wolffi | 85 | 99 | - |
| 56655 | *L.borgpetersenii* | Mini | - | - | - |
