# Supplementary material for: Identification of three extra-chromosomal replicons in Leptospira pathogenic strain and development of new shuttle vectors
Source: BMC Genomics. 2015 Feb 15;16(1):90. doi: 10.1186/s12864-015-1321-y (PMC4338851; doi:10.1186/s12864-015-1321-y)
Supplement: Additional file 2: — Characterization of genome DNA from the three plasmids by agarose gel electrophoresis and Southern blotting. [file 12864_2015_1321_MOESM2_ESM.docx]

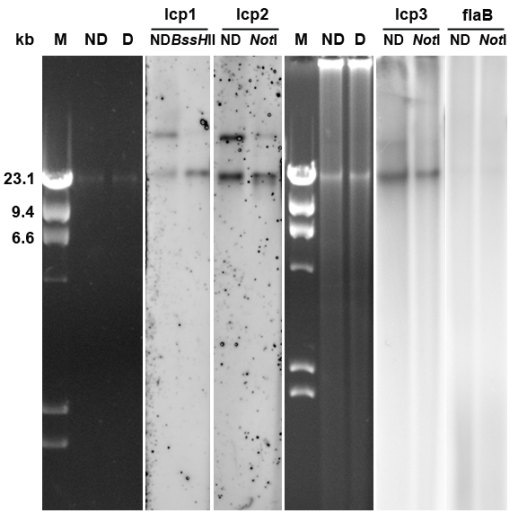


**Additional file 2. Characterization of genome DNA from the three plasmids by agarose gel electrophoresis and Southern blotting.** M, λ *Hind*III, bacteriophage λ-*Hind*III digest DNA Marker with a largest ladder 23.1kb; ND, undigested plasmid extraction; D, plasmid extraction digestion with restriction enzyme (*BssH*II, *Not*I). Southern blot analysis was performed with three plasmids specific probes (lcp1-probe, lcp2-probe and lcp3-probe) and chromosome gene *flaB* specific probe (flaB-probe) as the negative control. Primers for synthetize probes were listed in **Additional file 1, Table S3**. Notably, two bands appeared for these plasmids after agarose gel electrophoresis, with upper one predicted to be nicked open circular plasmid DNA while the lower one to be linear plasmid DNA. The plasmid extraction used for detection plasmids lcp1 and lcp2 was from different batches with that used for detection plasmid lcp3 and chromosome.
