# Supplementary material for: Identification of three extra-chromosomal replicons in Leptospira pathogenic strain and development of new shuttle vectors
Source: BMC Genomics. 2015 Feb 15;16(1):90. doi: 10.1186/s12864-015-1321-y (PMC4338851; doi:10.1186/s12864-015-1321-y)
Supplement: Additional file 3: — Stability of the three plasmids within the L. interrogans strain 56609. [file 12864_2015_1321_MOESM3_ESM.docx]

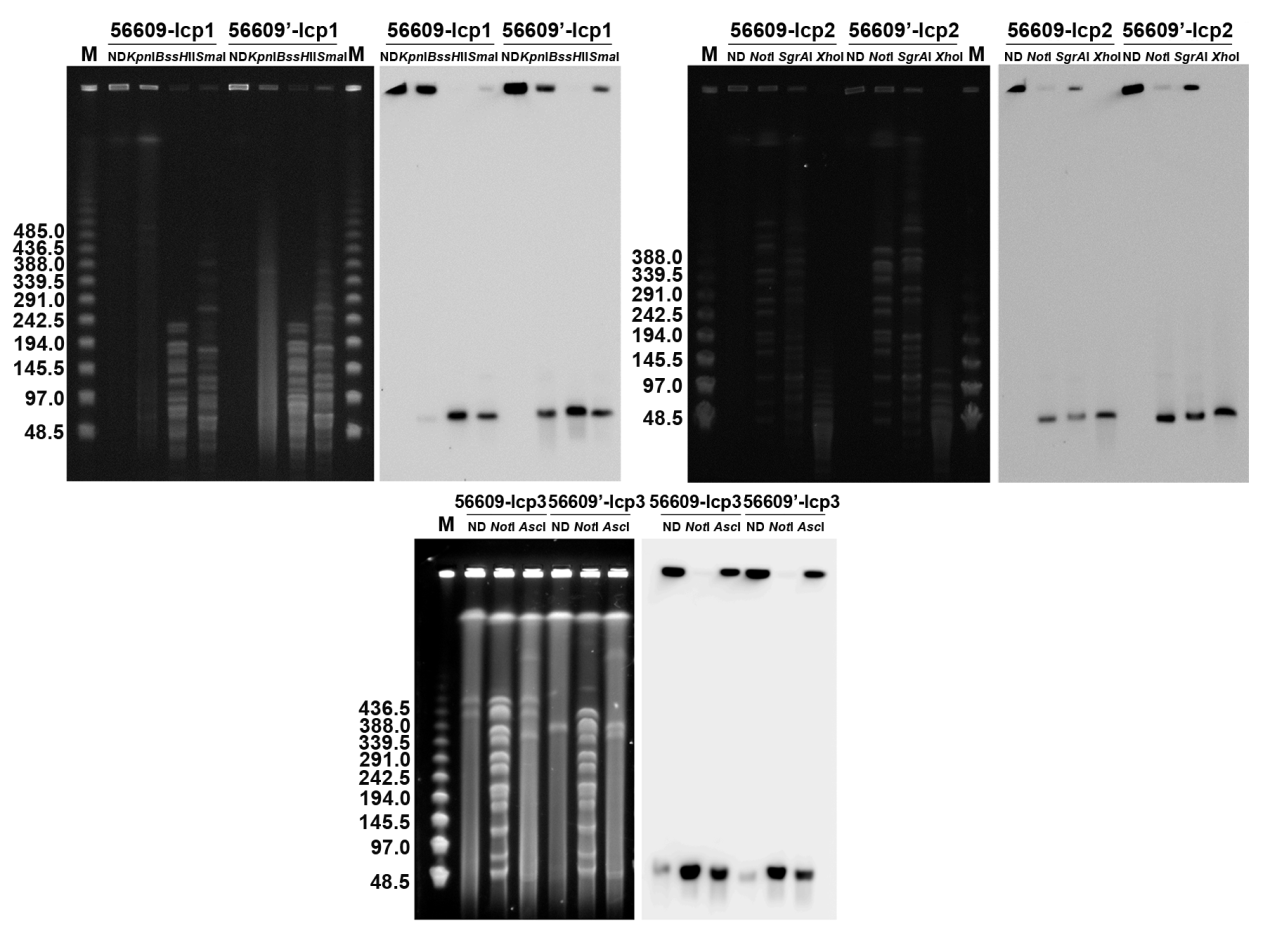


**Additional file 3. Detection of three plasmids stability within *L. interrogans* str. 56609.** Whole genomic analysis of *L. interrogans* str. 56609 and 56609’ (56609 after prolonged *in vitro* laboratory passage) by PFGE. ND, undigested DNA; M, the bacteriophage λ DNA multimer marker (monomer =48.5 kb). *In situ* bacteria cells were digested by different enzyme (*Asc*I, *Not*I, *Kpn*I, *BssH*II, *Sma*I, *SgrA*I, *Xho*I). For southern blot analysis, the genomic DNA was hybridized by the same probes as above.
