# Supplementary material for: Identification of three extra-chromosomal replicons in Leptospira pathogenic strain and development of new shuttle vectors
Source: BMC Genomics. 2015 Feb 15;16(1):90. doi: 10.1186/s12864-015-1321-y (PMC4338851; doi:10.1186/s12864-015-1321-y)
Supplement: Additional file 4: — RT-PCR and qPCR to detect gene expression after induction. [file 12864_2015_1321_MOESM4_ESM.docx]

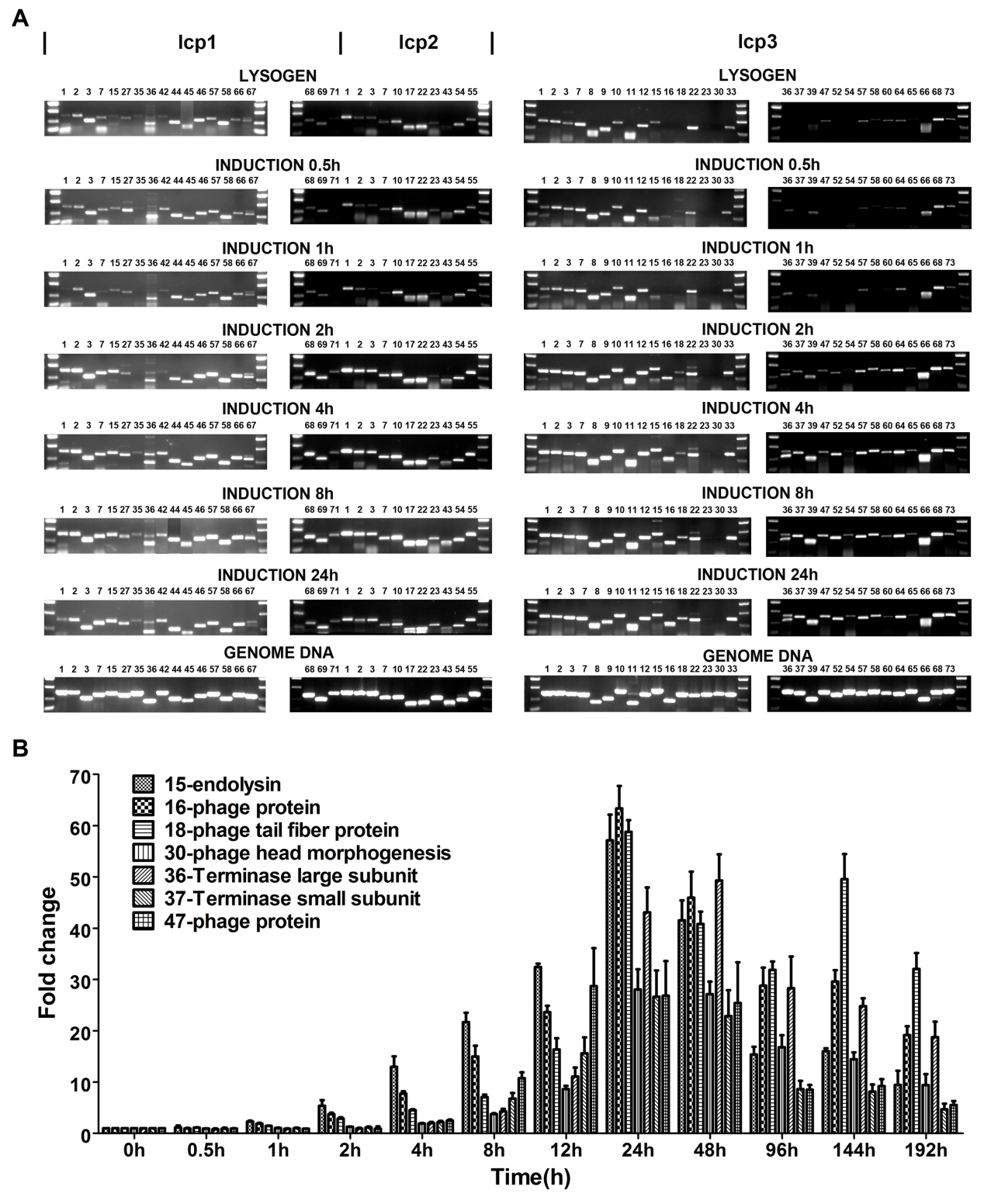


**Additional file 4. RT-PCR and qPCR to detect gene expression after induction.** (A) Transcription analysis of three plasmids by RT-PCR. The numbers of genes showed a selection of ORFs used for RT-PCR in three plasmids. Seven time points including lysogen, induction early steps and late steps of lytic stage for which mRNA was extracted were indicated in the panels. Genome DNA was used as a positive control. (B) qPCR of seven phage related genes in plasmid lcp3 with different induction time. Fold change was reported relative to *flaB*, a *L. interrogans* internal control. The experiments were performed in triplicate.
