# Supplementary material for: Identification of three extra-chromosomal replicons in Leptospira pathogenic strain and development of new shuttle vectors
Source: BMC Genomics. 2015 Feb 15;16(1):90. doi: 10.1186/s12864-015-1321-y (PMC4338851; doi:10.1186/s12864-015-1321-y)
Supplement: Additional file 5: — Identification of the three plasmids in 15 Chinese epidemic Leptospira strains and the saprophytic L. biflexa serovar Patoc strain Patoc I. [file 12864_2015_1321_MOESM5_ESM.docx]

**
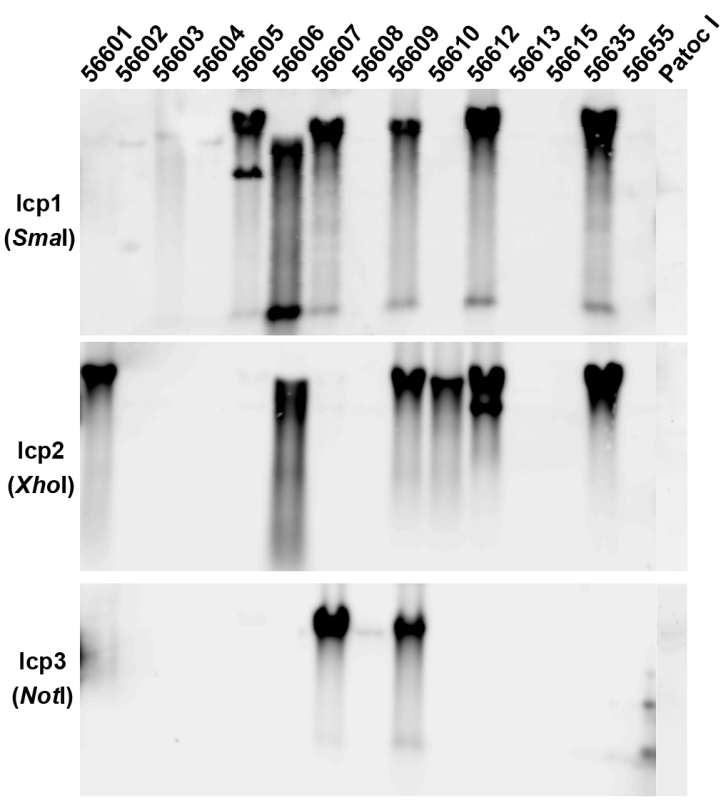
**

**Additional file 5. Determination of three plasmids in 15 Chinese epidemic Leptospira strains and saprophytic *L. biflexa* serovar Patoc strain Patoc 1.** Genomic DNA of 15 Leptospria strains was digested with three restriction enzymes (*Sma*I, *Xho*I, *Not*I), seperated on an agarose gel, blotted to a nylon membrane and then hybridized with the radiolabeled probes (corresponding to the *rep* genes of three plasmids). Probes were generated by PCR of T-vector plasmid DNA containing *rep* genes with primer pairs lcp1-rep-probe FR, lcp2-rep-probe-FR and lcp3-rep-probe-FR, respectively **(Additional file 1, Table S3)**.
